# Warning Color Changes in Response to Food Deprivation in the Pipevine Swallowtail Butterfly, *Battus philenor*

**DOI:** 10.1673/031.013.11001

**Published:** 2013-10-22

**Authors:** Kimberly V. Pegram, Alexandra C. Nahm, Ronald L. Rutowski

**Affiliations:** 1 School of Life Sciences, Arizona State University, Tempe, AZ 85287-4601; 2 Department of Biological Sciences, University of Maryland Baltimore County, Baltimore, MD 21250

**Keywords:** aposematic, coloration, food restriction, iridescence, pigment-based coloration

## Abstract

Predation on distasteful animals should favor warning coloration that is relatively conspicuous and phenotypically invariable. However, even among similarly colored individuals there can be variation in their warning signals. In butterflies, individual differences in larval feeding history could cause this variation. The warning signal of the pipevine swallowtail butterfly, *Battus philenor* L. (Lepidoptera: Papilionidae) consists of both a blue iridescent patch and pigmentbased orange spots on the ventral hindwing. *B. philenor* males also display a dorsal surface iridescent patch that functions as a sexual indicator signal. A previous study of iridescence in *B*. *philenor* found that the iridescent blue on both the dorsal and ventral hind wings is variable and significantly different between lab-reared and field-caught individuals. These differences could be the result of larval food deprivation in the field. Through experimental manipulation of larval diet, larval food deprivation was evaluated as a potential cause of the differences observed between lab and field individuals, and if food deprivation is a source of inter-individual variation in warning signals. *B. philenor* larvae were food restricted starting at two points in the last larval instar, and one group was fed through pupation. Adult coloration was then compared. Food deprivation led to poorer adult condition, as indicated by lower adult body mass, forewing length, and fat content of stressed individuals. As the level of food deprivation increased, the hue of the iridescent patches on both the dorsal and ventral hind wing shifted to shorter wavelengths, and the chroma of the orange spots decreased. The shifts in iridescent color did not match the differences previously found between lab and field individuals. However, the treatment differences indicate that food deprivation may be a significant source of warning color variation. The differences between the treatment groups are likely detectable by predators, but the effect of the variation on signal effectiveness and function remains to be empirically explored.

## Introduction

Warning coloration can function as a signal to predators of a prey's unprofitability (Poulton 1890; Cott 1940). Such signals are expected to be naturally selected by predation to facilitate learning and recognition by relevant predators (Guilford and Dawkins 1991; Ruxton et al. 2004) through increased conspicuousness (Gittleman and Harvey 1980; Gamberale-Stille and Tullberg 1999; Lindström et al. 1999; Riipi et al. 2001; Lindstedt et al. 2008) and/or reduced phenotypic variation (Guilford and Dawkins 1993; Mappes and Alatalo 1997; Beatty et al. 2004; Rowland et al. 2007). Either of these processes should lead to a reduction in genetic variation or the extent to which the structures that produce a warning signal respond to environmental variation during development. Nonetheless, warning colors often display surprising levels of interindividual variation in a population (Brakefield 1985; Brakefield and Liebert 1985; Holloway et al. 1995; Grill and Moore 1998; Grill 1999; Ojala et al. 2007; Lindstedt et al. 2008; Borer et al. 2010; Rutowski et al. 2010). This indicates that in the face of stabilizing selection there are factors that maintain variation in warning signal expression (Endler and Mappes 2004; Ojala et al. 2007; Sandre et al. 2007; Maan and Cummings 2008; Lindstedt et al. 2010; Lindstedt et al. 2011). One such potential factor in nature is variation among individuals in the extent to which they experience food restrictions during growth and development. The effects of food restriction or deprivation on sexual coloration are wellstudied (e.g., McGraw et al. 2002; Siefferman and Hill 2005; Kemp 2008; Punzalan et al. 2008), and reduced diet quality can affect warning coloration (e.g., Grill and Moore 1998; Grill 1999; Ojala et al. 2007), but the effects of food deprivation on warning coloration are unknown.

**Figure 1.**
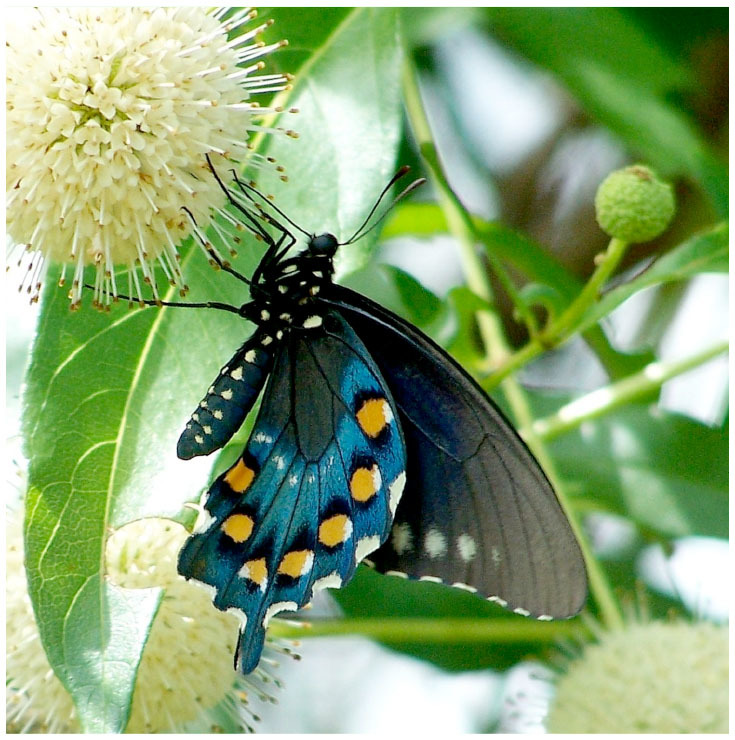
*Battus philenor* with ventral surface visible. High quality figures are available online.

Food deprivation in lepidopteran larvae is common in species that feed on small plants because they may have to travel between hostplants after complete defoliation of a plant (Stockhoff 1991; Fischer and Fiedler 2001). Larvae of the pipevine swallowtail butterfly, *Battus philenor* L. (Lepidoptera: Papilionidae) are especially susceptible to food deprivation during movement from one host plant to another. *B. philenor* larvae feed on plants in the genus *Aristolochia*, and individual hostplants rarely provide enough suitable foliage for complete larval development (Rausher 1979a, b, 1980; Rausher and Feeny 1980; Fordyce and Agrawal 2001), which can sometimes require more than 25 plants (Rausher 1980). The larvae also sequester aristolochic acids, which make them and the adults they produce unpalatable (Sime et al. 2000; Fordyce et al. 2005). To advertise this defense, adults display warning coloration on the ventral hind wing surface (Brower 1958; Codella and Lederhouse 1990), which consists of orange spots in a field of iridescent blue (Figure 1). Both colors are used by predators in recognizing *B. philenor* as distasteful (Pegram et al. 2013).

In Arizona, the iridescent warning coloration of *B. philenor* on the ventral hind wings varies in ways that may be attributed to differences in larval diet. Rutowski et al. (2010) reported significant differences in the iridescent coloration between lab-reared individuals fed *ad libitum* and field-caught individuals. Therefore, variation in larval food availability could be a source of adult color variation, including warning color variation. As in the *B. philenor* populations previously studied, populations in Arizona are likely to experience food deprivation as larvae because their hostplant, *Aristolochia watsonii* Wooton and Sandley (Aristolochiales: Aristolochiaceae), is a small plant that larvae often completely denude of leaves before completing development (personal observation). Therefore, to evaluate the role of food restriction on warning coloration and to determine if observed natural variation in iridescent signals is due to food deprivation, the amount of food to which *B. philenor* larvae had access was varied among three different treatments. The adult coloration was compared among treatment groups. The effects of food deprivation were evaluated for three different color patches: the iridescent blue field and the orange spots of the ventral hind wing surface, which contribute to the warning signal, and the iridescent blue on the male dorsal hind wing. The male dorsal hind wing is a signal used by females, likely to assess either male quality or species identity (Rutowski and Rajyaguru 2013).

If food deprivation is a source of variation in the iridescent signals of *B. philenor*, it is predicted that food deprivation will cause increased brightness, shorter wavelength hues, and higher chroma for the ventral surface iridescence and higher intensities in the dorsal iridescence. These expectations are based on the difference between animals reared in the lab and those from the field reported in Rutowski et al. (2010). Additionally, if there are differences in the ventral surface iridescence and the orange spots between treatments, even if this variation does not match that found in Rutowski et al. (2010), it will indicate that food restriction can be a significant source of intraspecific variation in warning signals.

## Materials and Methods

### Rearing conditions and study of developmental time course

Early instar *B. philenor* larvae and eggs were collected in the field near the confluence of Mesquite Wash and Sycamore Creek in central Arizona, USA (N 33° 43.784′, W 111° 30.997′). After collection, the animals were reared in an environmental chamber in which relative humidity was held at 55%. Temperature and light varied on a 16:8 L:D cycle, in which the temperature was 30° C with lights on for 16 hr and 24° C with the lights off for 8 hr. Larvae were fed cuttings from fieldcollected host plant, *A. -watsonii, ad libitum* until treatment began. These conditions and the field site are those described in Rutowski et al. (2010).

In order to develop a protocol of food restriction the time course of development for *B*. *philenor* larvae from the source population was determined. To determine the number, duration, and growth rate of larval instars, 10 larvae were raised as above, and their body mass was measured every day from hatching to pupation. The timing of molts in the last two instars was determined by putting a spot of paint on the integument and noting when it disappeared (i.e., had been shed with the exoskeleton). Earlier instar larvae were generally not marked because of the difficulty of doing so without injury, but four larvae were marked through their entire development and revealedthat there are five larval instars. Data on body condition and adult coloration of these individuals were not used in the analysis.

### Food deprivation treatment

Prior to the molt into the final larval instar, each larva was marked on the posterior dorsum with a small dot of green paint, placed in an individual cup with host plant, and randomly assigned to a treatment group. Because larvae and eggs were collected from many different plants in the field and across a 19-day-timespan, it is unlikely that they were closely related. Larvae were checked each day between 11:00 and 15:30. When the dot was no longer present on a larva, it indicated that it had molted into the 5th instar within the last 24 hours, and that day was set as Day 0 for that larva.

The final larval instar was chosen as the best developmental stage to restrict food availability because at this stage larvae consume the most host plant and are most likely to need to seek new plants in the field. Also, during the 5th instar they will likely attain a threshold mass above which they are able to pupate without additional food, as has been documented for other butterflies (Nijhout 1975; Jones 1976). After this threshold stage, larvae will not die of starvation if they do not find another host plant, but if food is available they will continue to feed and so may accrue additional resources that will be available during metamorphosis to produce morphological features, such as wing colors.

When each larva reached the 5th instar, it was placed into one of three treatment groups. In all three groups, larvae were fed *ad libitum* until food deprivation began. In the Day 3 treatment, larvae (n = 27) were given no food from the third day of the final larval instar until they pupated. This was the treatment groupthat experienced the highest level of food restriction. In the Day 4 treatment, food deprivation began on the fourth day of the last larval instar and continued until they pupated (n = 32). Deprivation in both groups began between 11:00 and 15:30 on the appropriate day. Larvae in the unrestricted treatment (n = 32) were provided with food *ad libitum* until they pupated. The third and fourth days of the final larval instar were chosen to begin food deprivation because no individual deprived of food from the second day or earlier survived to pupate (n = 5), and by the fifth day most larvae stopped feeding or otherwise started preparing for pupation.

Pupae were kept in the environmental chamber under the conditions described above until the adults eclosed, typically after 11–14 days. Individuals that had apparently entered diapause (n = 6), as indicated by the pupal stage lasting longer than 21 days, were not included in the analysis. Treatment did not have any apparent effect on whether they entered diapause, as there were two from each treatment. Upon eclosion, butterflies were placed in a refrigerator at 4° C for 24 hr and then freezekilled.

### Analysis of adults

Each adult's forewing length (wing tip to anterior point of wing insertion in the thorax) was measured using digital calipers to the nearest 0.01 mm. After removing the hind wings for mounting, the bodies were lyophilized for 24 hr, and the “dry” mass of each was measured to the nearest 0.1 mg. In addition, fat content of the bodies and forewings was measured as a percent of dry mass using the methods described in Fordyce and Nice (2008).

The hind wings were mounted on double-ply, museum-quality, black cardstock using photo mount adhesive. The right hind wing was mounted dorsal side up, and the left hind wing was mounted ventral side up. Reflectance spectra were obtained relative to an MgO white standard, using an USB 2000 spectrophotometer with a PX2 xenon light source and OOIBase 32 software (all from Ocean Optics, www.oceanoptics.com). The ventral iridescent reflectance was measured from an area proximal to the orange and black spot in the cell between M_3_ and Cu_1_ veins on the hind wing (Borror et al. 1992), and the dorsal reflectance was measured in the same cell from an area proximal to the white spot on the hind wing. The angle between the light beam and the optical axis of the collector was 60° with the wing positioned so that its base pointed toward the light beam. The specimen was tilted around an axis perpendicular to the long axis of the wing until the reflectance of the iridescence around 500 nm was highest (see Figure 2 in Rutowski et al. 2010). Maximizing the iridescence of each wing in this manner allowed for consistent reflectance measurements, as some individuals may vary in the exact angle that produces peak reflectance (Kemp and Rutowksi 2007; Kemp 2008; Meadows et al. 2011) and measurements that are comparable to previous analyses of *B*. *philenor* iridescent coloration (Rutowski et al. 2010; Rutowski and Rajyaguru 2013). The orange spot reflectance was measured on the most anterior orange spot, with the collector and light beam positioned the same as for the iridescent patch measures and the wing surface perpendicular to bisector of the angle between collection and light beam.

**Figure 2.**
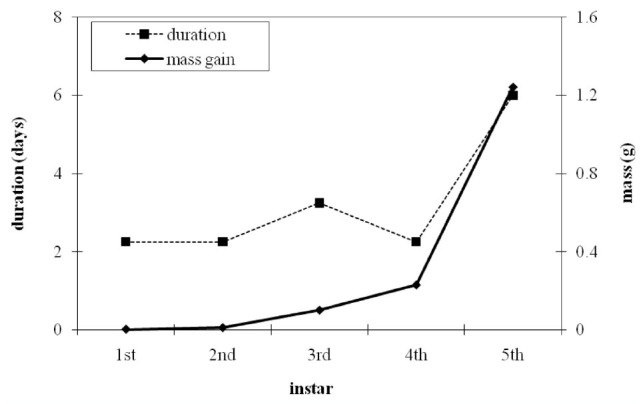
Mass gain and duration for each larval instar obtained by weighing *Battus philenor* larvae every day and recording molts (n = 10). Approximately 75% of total larval mass gain occurs in the final larval instar. The final instar lasts an average of 6 days. High quality figures are available online.

Each mounted hind wing was measured twice, in two separate rounds. All pairs of measurements on the same spot were within 5% of each other. The color parameters obtained from these two measurements were then averaged for the analysis. Only reflectance from 300–700 nm was used in the analysis. Spectra were characterized using three color parameters: brightness, hue, and chroma. Brightness is the amount of light reflected from the spot measured, and for both the orange and blue was calculated as the average percent reflectance from 300–700 nm (Montgomerie 2006). Hue identifies the wavelengths in which the most light is reflected and therefore contributes to the perceived color of the signal. Chroma (also known as saturation) is the spectral purity of the reflected light (Montgomerie 2006). Hue and chroma needed to be calculated differently for the iridescent blue and the diffusely reflecting orange because the orange reflectance lacks a clear peak. For the iridescent reflectance, hue is the wavelength at which the maximum reflectance is observed, and chroma is the summed reflectances between 50 nm above and below the peak wavelength, divided by the summed reflectances between 300 and 700 nm (Rutowski et al. 2010). For the reflectance of the orange spot, hue is the wavelength at which the percent reflectance is halfway between the minimum and maximum reflectance (Morehouse and Rutowski 2010) over all wavelengths (300–700 nm). Chroma is the difference between the minimum and maximum reflectance, divided by the average reflectance over the 300–700 nm range (Montgomerie 2006).

To infer whether avian predators would be able to distinguish differences in the warning colors between the different treatment groups, AVICOL version 6 (Gomez 2006) and a physiological visual model based on Vorobyev and Osorio (1998) were used. The model used the spectral sensitivities and cone proportions for Blue Tits, *Cyanistes caeruleus*, a well understood passerine visual system (Hart et al. 2000). While Blue Tits are not predators of *B*. *philenor*, the visual sensitivities of birds are not highly variable (Hart 2001), and the specific visual sensitivities of known *B. philenor* predators (e.g., Cactus Wrens, *Campylorhynchus brunneicapillus*, and Curve-billed Thrashers, *Toxostoma curvirostre*) are not known. As such, the visual sensitivities of Blue Tits are a good surrogate of those of predators of *B. philenor*. The ambient light was a measure of downwelling irradiance from the field site, measured with the USB 2000 spectrophotometer described earlier fitted with a cosine-corrected probe on the end of the collector fiber. The model was used to obtain the chromatic contrasts between all three treatments for both the ventral orange spots and ventral iridescence. Chromatic contrast is reported in units of just noticeable differences (jnd), and a difference above 1 jnd is considered to be distinguishable by a bird (Vorobyev et al. 1998).

### Statistics

MANOVAs were used to determine the over-all effects of treatment on body condition (i.e., dry body mass, forewing length, and fat content) and the color parameters (i.e., hue, chroma, and brightness) of the two ventral color components. Each model included sex, treatment, and sex by treatment interaction as the factors. To sort out the effects of the individual variables in the significant MANOVAs, univariate ANOVAs and the standardized coefficients from discriminant analysis were used. If these tests revealed a significant treatment effect for a variable, the significance of differences in that variable between specific treatment groups was assessed using Tukey's posthoc tests. Three mixed models with restricted maximum likelihood were used to determine the response of male iridescence (i.e., brightness, hue, and chroma of both iridescent surfaces) and included surface, treatment, and surface by treatment interaction as fixed effects and individual as a random effect. For these models, Bonferroni corrections using α = 0.05 requires *p* ≤ 0.016 for a factor to be considered significant. All statistical analyses were run using SPSS version 19 (IBM, www.ibm.com) with a 0.05 level of significance where not otherwise indicated. Tests for normality revealed that brightness measurements were not normally distributed, and so those measurements were log transformed before analysis. Dependent variables in all models had equal variance.

## Results

### Study of developmental time course

Under the rearing conditions, *B. philenor* larvae from the Arizona population that was studied (1) have five instars, (2) acquire 75% of their maximum larval body mass during the 5th instar, and (3) spend an average of six days in the 5th instar before pupating (Figure 2). These results guided the design of the treatments. Interestingly, *B. philenor* larvae in other geographic areas have six instars (J. Fordyce, personal communication), which indicates that *B. philenor* may be another case of intraspecific variability in number of instars (e.g., Esperk et al. 2007).

### Effects of food stress on body condition and pupation time

Treatment had an overall effect on body condition (MANOVA: Wilk's λ = 0.353, *F*_6,154_ = 17.29, *p* < 0.001). Univariate ANOVAs revealed that treatment negatively affected adult dry body mass, forewing length, and fat content, and the highest level of food restriction produced smaller adults with a lower percentage of fat in their bodies (Table 1). Standardized discriminant coefficients indicated that forewing length explains more of the variance due to treatment than body mass or fat content. The dry body mass of adults in the Day 3 treatment (highest level of food restriction) was only 50–55% that of animals with unrestricted access to food (Table 1). Forewing length of males and females in the Day 3 treatment was reduced by 13% and 12%, respectively, compared to those in the Unrestricted group (Table 1). For fat content (as a percentage of body mass), Tukey's comparisons of differences among treatment groups revealed that the adults in the Unrestricted treatment had a significantly higher fat content than those in the Day 3 treatment (*p* = 0.017) and in the Day 4 treatment (*p* = 0.011). Fat content of the adults in the Day 3 and Day 4 treatments was not significantly different (*p* = 0.999).

**Table 1.**
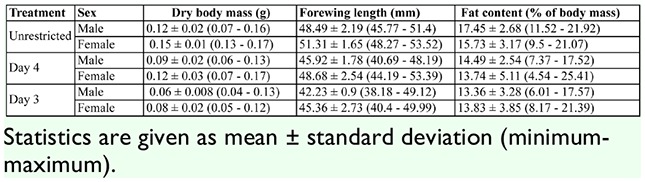
A summary of dry body mass, forewing length, and fat content values of *Battus philenor*.

**Table 2.**
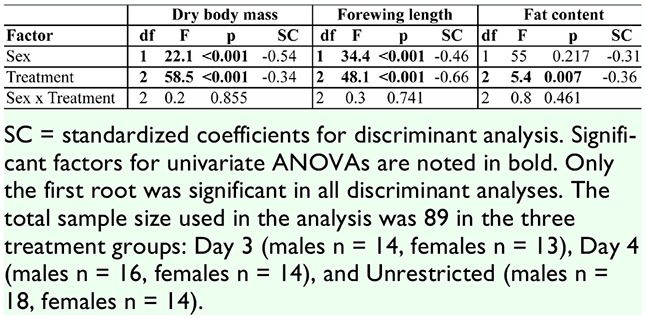
Results of univariate tests and discriminant analysis for body condition measures of *Battus philenor*.

The sexes differed significantly in body size and fat content (MANOVA: Wilk's λ = 0.693, *F*_3,76_= 11.24, *p* > 0.001). In univariate ANOVAs, sex was a significant factor for both body mass and forewing length (Table 2); *B*. *philenor* males are smaller than females. However, there was no significant effect of sex on fat content (Table 2). In the discriminant analysis, males and females differed in all three measures of body condition, but mass and forewing length had the highest coefficients (Table 2). There was no sex by treatment interaction for the three measures of body condition (MANOVA: Wilk's λ = 0.940, *F*_6,152_= 0.192, *p* = 0.577).

Duration of the pupal stage (measured in days) was significantly affected by treatment, with food-restricted individuals spending less time in the pupal stage (Kruskal-Wallis χ^2^ = 12.67, df = 2, *p* = 0.002). The mean duration of the pupal stage was 13.3 days for the Unrestrcited treatment group, 13.1 days for the Day 4 treatment groups, and 12.7 days for the Day 3 treatmentgroup.

### Effects of food deprivation on warning coloration

**Figure 3.**
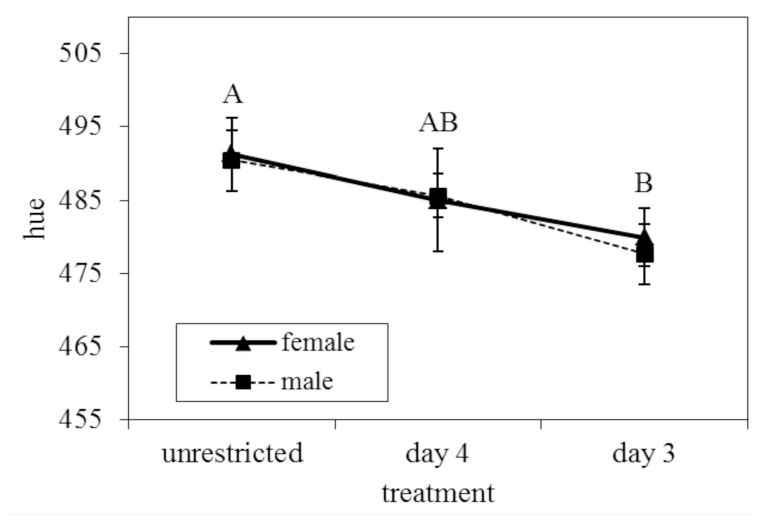
Hue measured on ventral iridescence of *Battus philenor* as the wavelength of highest reflectance. There was a shift towards shorter wavelengths in hue on both iridescent surfaces (dorsal not shown) with higher food restriction. Bars represent one standard error. Different letters represent significantly different values. Shared letters indicate responses are not significantly different. High quality figures are available online.

For the ventral iridescence, there was a significant effect of food deprivation treatment (MANOVA: Wilk's λ = 0.826, *F*_6,162_ = 2.71, *p* = 0.016). Both discriminant analysis and univariate tests revealed that treatment affected hue the most (Table 3). Tukey's post-hoc comparisons revealed a significant shift in hue towards shorter wavelengths (towards blue) with increasing food restriction (Table 3), shifting almost 15 nm between adults in the Unrestricted and Day 3 treatments (Table 4; Figure 3). The hue of the adults in the Day 3 treatment was significantly bluer than those of the Unrestricted treatment (*p =* 0.028) but was not different from those in the Day 4 treatment (*p* = 0.340). Univariate ANOVAs did not reveal an effect of food restriction on brightness or chroma (Table 3). Additionally, there was no difference between the ventral iridescence of the sexes (MANOVA: Wilk's λ = 0.939, *F*_3,81_ = 1.75, *p* = 0.164) and no sex by treatment interaction (MANOVA: Wilk's *λ* = 0.972, *F*_6,162_ = 0.393, *p* = 0.883).

**Table 3.**
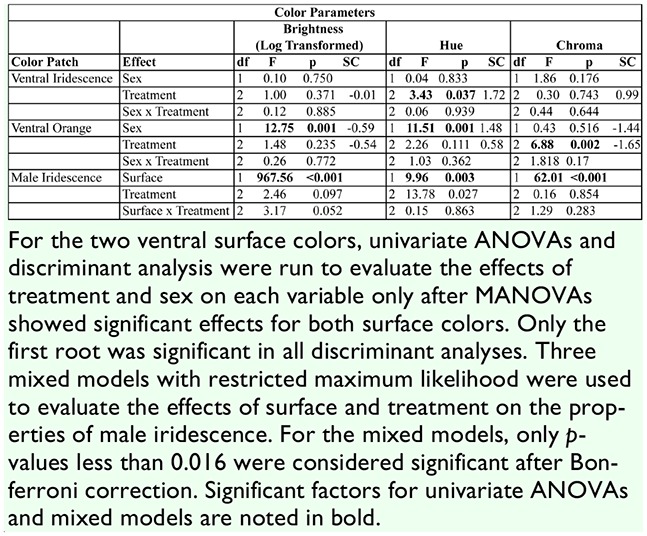
Results of univariate tests and discriminant analysis (SC, standardized coefficients) for color parameters of *Battus philenor*.

**Table 4.**
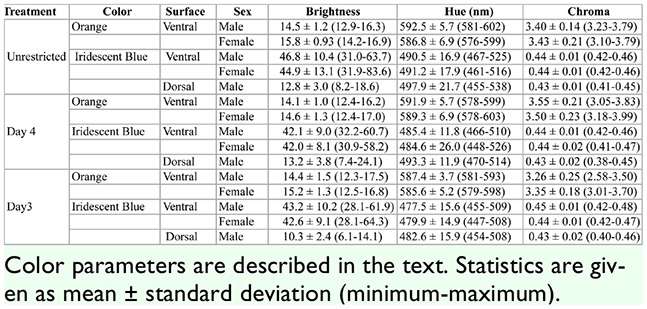
A summary of color parameter values obtained from reflectance spectra for all treatment groups of *Battus philenor*.

**Figure 4.**
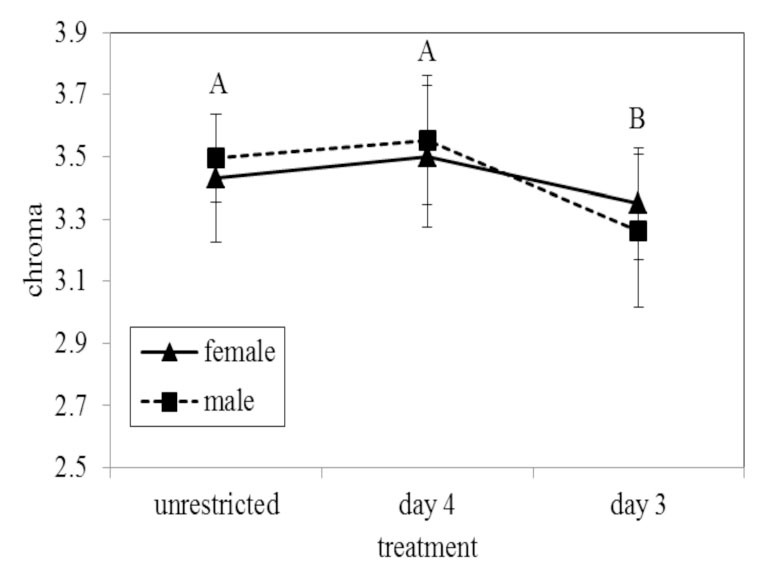
Mean chroma of ventral orange spots from reflectance spectra on *Battus philenor*. Chroma was measured as the difference between the minimum and maximum reflectance divided by the average reflectance over 300–700 nm. Bars represent one standard error. Different letters represent significantly different values. Shared letters indicate responses are not significantly different. High quality figures are available online.

Treatment also significantly affected the ventral orange spots (MANOVA: Wilk's λ = 0.671, *F*_6,148_. 148 = 5.45, *p* < 0.001). Univariate tests and the standardized coefficients revealed that chroma was the color parameter most affected by treatment (Table 3). Chroma decreased with increasing food deprivation (Table 4). Tukey's post-hoc comparisons revealed that the adults in the Day 3 treatment were significantly less chromatic than those in the Day 4 (*p* = 0.002) and Unrestricted treatments (*p* = 0.019). Individuals in the Day 4 treatment were not significantly different in chroma from those in the Unrestricted treatment (Figure 4). In addition to the effect of treatment, there was also a significant effect of sex on the ventral orange spots (MANOVA: Wilk's λ = 0.652, *F*_3,74_ = 13.17,*p* < 0.001). Univariate ANOVAs and discriminant coefficients revealed that sex mostly influenced brightness and hue (Table 3). On average, females’ orange spots were brighter than males’ and had a lower (shorter wavelength) hue than males’ (Table 4). There was also no sex by treatment interaction for the ventral orange (MANOVA: Wilk's λ = 0.929, *F*_6,148_ =0.928, *p* = 0.477).

Models of avian color vision suggest that warning color differences between treatment groups would be distinguishable by an avian predator. For the ventral orange, the chromatic contrast between the color of the adults from the Unrestricted treatment and the Day 4 treatment was 5.76 jnd, between the Unrestricted treatment and the Day 3 treatment was 5.87 jnd, and between the Day 3 treatment and Day 4 treatment was 9.56 jnd. For the ventral iridescence, the chromatic contrast between the adults of the Unrestricted treatment and the Day 4 treatment was 2.96 jnd, between the adults of the Unrestricted treatment and the Day 3 treatment was 4.53 jnd, and between the adults of the Day 3 treatment and the Day 4 treatment was 1.59 jnd. Because all of these chromatic contrasts were above 1 jnd, they were all considered to be distinguishable by a bird.

### Comparison of two iridescent surfaces

The statistical analysis on the color measurements for the iridescent areas from both male wing surfaces did not reveal an effect of treatment after correcting for multiple mixed model comparisons. However, Tukey's posthoc comparisons revealed differences between the treatment groups in hue, where hue decreased (shifted towards blue) with increasing food restriction on both surfaces. Unrestricted individuals were significantly different from Day 3 individuals (*p* = 0.008) but not Day 4 individuals (*p* = 0.320). Day 4 individuals were also not significantly different from Day 3 individuals (*p* = 0.068). There was no significant surface by treatment interaction to suggest the two surfaces responded differently to food deprivation (Table 3). Also, all color parameters were significantly different between the two iridescent surfaces (Table 3). The ventral iridescence of males is brighter, has a lower hue, and is more chromatic than the dorsal surface. The difference in brightness and chroma were expected from the results reported in Rutowski et al. (2010). The variance accounted for by individual was significant for hue (Wald Z = 3.269, *p* = 0.001) and chroma (Wald *Z* = 3.211,*p* = 0.001), suggesting correlations between the two surfaces in these parameters.

## Discussion

### Measures of body condition

Compared to those with unrestricted access to food during development, food-restricted larvae developed into significantly smaller adults with smaller fat reserves. This result was expected based on previous studies of larval food limitation in butterflies (e.g., Bauerfeind and Fischer 2005; Boggs and Freeman 2005). The size of the adults produced from Day 3 treatment larvae was within the range of adult sizes observed in the field (Rutowski et al. 2010), indicating that the level of food deprivation induced in the Day 3 treatment was likely within the range of food limitation that this species experiences in nature. The effect of larval food deprivation on the level of sequestered aristolochic acids is currently under investigation.

### Warning coloration

Food restriction produced significant variation in both the orange and blue components of the ventral warning coloration of *B. philenor*. The hue of the ventral blue iridescence shifted toshorter wavelengths with increased food deprivation. The chroma of the orange spots decreased with increased food deprivation.

These results suggest proximate links between coloration, the structures and chemicals that produce color, and diet quantity, but these linkages are not clear at the moment (see Kemp et al. 2006). For *B. philenor*, this is true for both the orange spots and the blue patches, but there are some possible connections that could be tested. The diffuse reflection of the orange spots indicates that the pigments played a major role in shaping the reflectance spectrum by absorbing short wavelengths, which allows longer wavelengths to be reflected from the wing surface (Rutowski et al. 2005). The specific pigments involved are not known but are likely to be ommachromes or papilochromes synthesized de novo by the butterflies from the amino acid tryptophan (Nijhout 1991). The chroma of the orange spots should be positively related to the quantity of pigment in the scale, as more pigment means greater absorption of short wavelength light. During development, diet-restricted individuals whose orange is less chromatic may deposit less pigment in their scales due to a lower availability of tryptophan. On the other hand, the iridescent blue is likely a product of thin film interference, and the higher hue of diet-restricted individuals suggests a thicker film (Land 1972). If true, it is not clear how the film would be thicker in diet-restricted individuals who presumably experience restrictions in the materials needed to build these cuticular films. Again, questions about the potential proximate connections between diet and color phenotype remain untested but warrant investigation.

An experiment with captive Curve-billed Thrashers showed that the blue iridescence and the orange spots of the ventral hind wingwere used by avian predators to recognize *B*. *philenor* as distasteful, and each component alone elicited a rejection response (Pegram et al. 2013). It is unknown whether the variation in the hue of the iridescent patches induced by food restriction would alter the effectiveness of the aposematic coloration of *B. philenor*. Although both reptiles and invertebrates (e.g., spiders and dragonflies) have been observed preying on *B. philenor* (Rausher 1979b, 1980), insectivorous birds are likely to be their most common predators in Arizona (Pegram, Han, and Rutowski, unpublished data). Visual models indicated that birds should be able to distinguish the spectral differences observed in adult coloration due to treatment. However, even though avian predators may be able to discriminate these colors, predators may generalize a learned warning signal to similar colors (Ham et al. 2006; Ruxton et al. 2008; Svádová et al. 2009) or the differences may not be detectable in complex and changing conditions of lighting and background (Lindstedt et al. 2011). Either way, the color shifts caused by food deprivation may not decrease signal effectiveness. Signal effectiveness could also be influenced if the observed responses altered conspicuousness (Gittleman and Harvey 1980; Gamberale-Stille and Tullberg 1999; Lindström et al. 1999; Riipi et al. 2001; Lindstedt et al. 2008).From these results, it is concluded that food deprivation did contribute to intraspecific variation in warning coloration, but determining if this variation correlates with signal effectiveness will require further study.

### Response of iridescent coloration and comparison to natural coloration

The hue of the ventral and dorsal iridescent patches shifted to shorter (bluer) wavelengths with increased food deprivation. This was the opposite direction of what was predicted based on the results of Rutowski et al. (2010).Rutowski et al. (2010) also found that labreared individuals had more intense ventral iridescence than did field-caught individuals, where no effect of rearing conditions on ventral iridescence brightness was found in our study. Therefore, differences in the lab and field individuals previously observed were not likely due to increased food deprivation in the field-caught *B. philenor*. The difference between the dorsal and ventral surfaces in male chroma observed in our study was expected based on Rutowski et al. (2010), but the lack of treatment effects and differences between the sexes was inconsistent with their results. Differences between male and female ventral hue were observed in the previous study, but not in our study. From these differences, it can be concluded that the differences observed between lab and field individuals in Rutowski et al. (2010) were not likely caused by food deprivation in field individuals.

However, differences in the results of these studies could be caused by at least two other factors. First, there were differences between the studies in seasons in which observations occurred (spring (Rutowski et al. 2010) vs. autumn (our study)). Second, the larvae of the field-caught individuals could have undergone food deprivation throughout the larval stage, while the larvae in our experiment only underwent food restriction in the last larval instar.

Also, the hue and chroma of an individual's dorsal iridescence were correlated with the hue and chroma of its ventral wing surface, which suggests a coupling of the iridescent surfaces. This is consistent with the findings of Rutowski et al. (2010), but the causes of this coupling are not understood at this time.

Because the dorsal coloration may serve as a signal of male quality (Rutowski and Rajyaguru 2013), we expected to see heightened condition dependence over a naturally selected signal (e.g., Andersson 1986; Cotton et al. 2004a, b; Kemp 2008). However, there were no significant surface by treatment interactions to suggest there is heightened condition dependence of the iridescence on the dorsal surface.

### Conclusions

Food deprivation can be a common ecological occurrence for lepidopteran larvae that feed on relatively small hostplants, like the *Aristolochia* plants used by some populations of *B. philenor* (Rausher 1979a, 1979b, 1980; Rausher and Feeny 1980; Fordyce and Agrawal 2001), and may affect the development of adult color signals. In the case of *B*. *philenor*, spectral properties of the iridescent blue patches and diffusely reflecting orange spots that act as warning signals changed in response to a food deprivation, suggesting that food deprivation during the larval stage can be a significant source of intraspecific variation in coloration. However, because the findings were not wholly consistent with differences between field-caught and lab-reared individuals reported in previous studies (Rutowski et al. 2010), there are likely to be additional factors that explain the variation in adult coloration. The consequences of any of this variation for signal function remain to be explored.
